# A Two-Minute Walking Test With a Smartphone App for Persons With Multiple Sclerosis: Validation Study

**DOI:** 10.2196/29128

**Published:** 2021-11-17

**Authors:** Pim van Oirschot, Marco Heerings, Karine Wendrich, Bram den Teuling, Frank Dorssers, René van Ee, Marijn Bart Martens, Peter Joseph Jongen

**Affiliations:** 1 Orikami Digital Health Products Nijmegen Netherlands; 2 Dutch National Multiple Sclerosis Foundation Rotterdam Netherlands; 3 Radboud University Medical Center Nijmegen Netherlands; 4 Faculty of Science Institute for Science in Society Radboud University Nijmegen Netherlands; 5 Sint Maartenskliniek Nijmegen Netherlands; 6 Drug Target ID Nijmegen Netherlands; 7 NeuroDrug Research BV Nijmegen Netherlands; 8 Department of Community & Occupational Medicine University Medical Centre Groningen Groningen Netherlands; 9 MS4 Research Institute Nijmegen Netherlands

**Keywords:** multiple sclerosis, relapsing remitting, mobility, mobile phone, 2-Minute Walking Test

## Abstract

**Background:**

Walking disturbances are a common dysfunction in persons with multiple sclerosis (MS). The 2-Minute Walking Test (2MWT) is widely used to quantify walking speed. We implemented a smartphone-based 2MWT (s2MWT) in MS sherpa, an app for persons with MS. When performing the s2MWT, users of the app are instructed to walk as fast as safely possible for 2 minutes in the open air, while the app records their movement and calculates the distance walked.

**Objective:**

The aim of this study is to investigate the concurrent validity and test-retest reliability of the MS sherpa s2MWT.

**Methods:**

We performed a validation study on 25 persons with relapsing-remitting MS and 79 healthy control (HC) participants. In the HC group, 21 participants were matched to the persons with MS based on age, gender, and education and these followed the same assessment schedule as the persons with MS (the *HC-matched* group), whereas 58 participants had a less intense assessment schedule to determine reference values (the *HC-normative* group). Intraclass correlation coefficients (ICCs) were determined between the distance measured by the s2MWT and the distance measured using distance markers on the pavement during these s2MWT assessments. ICCs were also determined for test-retest reliability and derived from 10 smartphone tests per study participant, with 3 days in between each test. We interviewed 7 study participants with MS regarding their experiences with the s2MWT.

**Results:**

In total, 755 s2MWTs were completed. The adherence rate for the persons with MS and the participants in the HC-matched group was 92.4% (425/460). The calculated distance walked on the s2MWT was, on average, 8.43 m or 5% (SD 18.9 m or 11%) higher than the distance measured using distance markers (n=43). An ICC of 0.817 was found for the concurrent validity of the s2MWT in the combined analysis of persons with MS and HC participants. Average ICCs of 9 test-retest reliability analyses of the s2MWT for persons with MS and the participants in the HC-matched group were 0.648 (SD 0.150) and 0.600 (SD 0.090), respectively, whereas the average ICC of 2 test-retest reliability analyses of the s2MWT for the participants in the HC-normative group was 0.700 (SD 0.029). The interviewed study participants found the s2MWT easy to perform, but they also expressed that the test results can be confronting and that a pressure to reach a certain distance can be experienced.

**Conclusions:**

The high correlation between s2MWT distance and the conventional 2MWT distance indicates a good concurrent validity. Similarly, high correlations underpin a good test-retest reliability of the s2MWT. We conclude that the s2MWT can be used to measure the distance that the persons with MS walk in 2 minutes outdoors near their home, from which both clinical studies and clinical practice can benefit.

## Introduction

### Background

Multiple sclerosis (MS) is a common and as yet incurable chronic neurological condition [1]. It affects the central nervous system, involving demyelination and resulting in impairment of the nerve conduction. MS symptoms are different for virtually every person with MS, and the course of the disease is unpredictable. Symptoms of MS include, among others, fatigue, cognitive impairment, visual disturbances, sensory disturbances, and balance problems. For some persons with MS, the disease progresses slowly, while others experience years of rapidly increasing disability soon after the diagnosis. MS is most commonly diagnosed in people in their 20s and 30s, although it can develop at any age. MS affects 3 times more women than men.

Approximately 75% of the persons with MS experience a clinically significant walking disturbance, which poses a barrier to being physically active [2]. Mobility problems may arise from various factors, such as fatigue, decreasing muscle strength, spasticity, and ataxia and may result in musculoskeletal pain in the back, hips, legs, and arms. Pain in turn may further reduce walking ability. Walking limitations are a key component of disability in persons with MS. The Expanded Disability Status Scale (EDSS), which ranges from 0 to 10 and is universally used by health professionals to quantify physical disability in persons with MS, relies on walking as the main measure of disability [3]. Physical activity is highly relevant for patients because it has a large impact on employment and generally on the quality of life [4]. Mobility supports social participation and, in many cases, the ability to work, which is important to prevent persons with MS feeling isolated and depressed [5].

Several walking tests are available for persons with MS, including the Timed Up and GoTest, the Timed 25-Foot Walk (T25FW), and 6-Minute Walking Test (6MWT).

The Timed Up and Go test measures the time that a person takes to rise from a chair, walk 3 m, turn around, walk back to the chair, and sit down. The T25FW measures the time that a person takes to walk 25 feet, approximately 7.6 m. In the 6MWT, the distance walked in 6 minutes is measured. Bennett et al [6] showed that the distance walked on a 2-Minute Walking Test (2MWT), a 3 times shorter variant of the 6MWT, highly correlates with Timed Up and Go, T25FW, and 6MWT scores and that it also correlates with EDSS.

Walking assessments for persons with MS can currently be scheduled upon request of their health care professionals. Usually, such an assessment is done in the MS clinic or at the physiotherapist. Recent technological advances show promise that in the near future, walking tests might be performed in the home environment of a person with MS [7-15]. This saves time and costs and makes it possible to schedule assessments more frequently. The wealth of data from regularly performed home assessments could also improve clinical decision-making because it provides health care providers with quantitative information about changes in their patients’ walking speed.

MS sherpa (Orikami Digital Health Products) is a software used as a medical device and intended to support the monitoring of persons with MS to give patients and their health care professionals personalized insight into the presence and progress of MS-related symptoms and signs [16-20]. MS sherpa is a system consisting of a smartphone app (supported on Android and iOS) for data collection and data presentation, a cloud service for data storage, analysis algorithms, and a clinician or researcher dashboard for user management and data presentation. The product is commercially available. More information can be found on the MS sherpa website [20].

It is possible to do a smartphone 2MWT (s2MWT) with MS sherpa. Instructions in an explanatory text in the app include that users should walk outside as fast as safely possible while still not running or jogging using a walking aid if necessary, with their phone in their trouser pocket during the test. Once an accurate GPS location signal is found, the test can be started. After the start button is pressed, users can place their smartphone in their trouser pocket during a 5-second countdown. At the end of the countdown, the users should start walking, and they can stop when they feel a vibration and hear a sound exactly 2 minutes later. Then, the distance walked is calculated and the test result is displayed in the app. The s2MWT differs in concept from traditional 2MWTs, which are generally performed indoors by walking on a level surface between 2 lines with a known distance, with various methods (and accuracies) for determining the length of the final stretch.

### Objective

The aim of this study is to investigate the concurrent validity and test-retest reliability of the s2MWT that is implemented in MS sherpa. The validation of the s2MWT was part of MS Self—a validation study during which participants performed self-monitoring assessments during 4 weeks with a precursor of MS sherpa, the *Mijn Kwik* (Orikami Digital Health Products) app and a Fitbit Charge 2 wearable. In particular, we investigated if the distance measured by the s2MWT agreed with the distance measured using distance markers on the pavement. Furthermore, we investigated the first experiences of persons with MS with digital self-monitoring through smartphone apps and activity trackers by interviewing 7 study participants with MS as part of MS Self [16]. In this paper, we present the interview results for the s2MWT.

## Methods

### Study Design

We recently reported all relevant details about the study design, including the inclusion criteria, information about the recruitment of study participants, ethical approval and informed consent, and data collection in a publication about the validity and test-retest reliability of the smartphone variant of the Symbol Digit Modalities Test—as an assessment tool for cognitive processing speed—that is implemented in MS sherpa [17]. In summary, the study was performed on 25 persons with relapsing-remitting MS and 2 groups of healthy control (HC) participants (n=79). The HC participants in the first control group (HC matched, n=21) were matched to the persons with MS with regard to age, gender, and education. The second control group (HC normative, n=58) was set up to determine the normal distribution for the smartphone test results. The app was installed on the study participants’ smartphones during the first day of the study.

**Figure 1:**
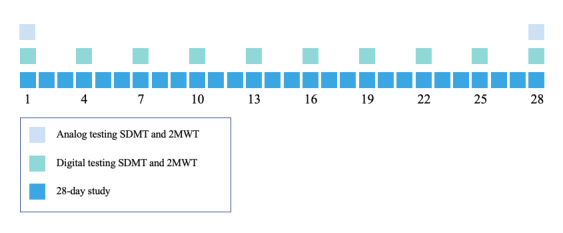
Overview of the study design and assessment scheme—the Symbol Digit Modalities Test (SDMT) and 2-Minute Walking Test (2MWT)—reproduced from van Oirschot et al [17].

Figure 1 shows a schematic overview of the study design for the persons with MS and the participants in the HC-matched group. In total, 10 home assessments were planned for the persons with MS and the study participants in the HC-matched group in 28 days, with 3 days in between each test. Before the start of MS Self, 10 of the 25 persons with MS were randomly contacted by mail for the qualitative part of the study. Interviews were scheduled with 7 participants before and after the study, resulting in 14 interviews. More information about the interview methods is published elsewhere [16].

**Figure 2:**
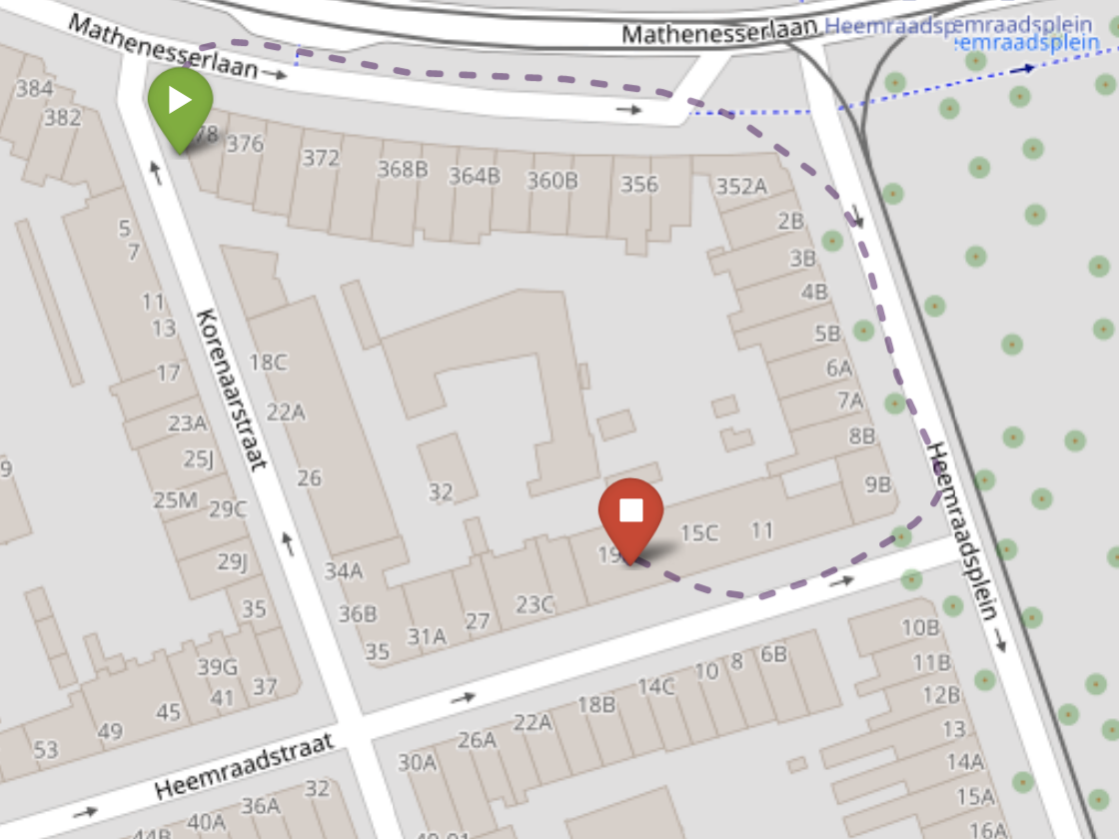
Street map of the block around the former premises of the Dutch National MS Foundation, Rotterdam, the Netherlands, on which the validation assessments took place. With a dashed line, the reconstructed path that was walked during one of the smartphone-based 2-Minute Walking Test is presented, starting from the green (play) marker and ending at the red (stop) marker.

The HC participants in the HC-normative group were instructed to perform the s2MWT 3 times in total, with 1 week in between the assessments. From these tests and from the 10 home assessments of the other study participants, the test-retest reliability of the s2MWT was determined. The concurrent validity of the s2MWT was determined from the comparison of the distance measured by the s2MWT with the distance measured using distance markers on the pavement during the s2MWT assessments—the 2MWTs that are described as *analog testing* in Figure 1. Hereafter, these assessments are referred to as the validation assessments. Note that there are 2 validation assessments per study participant: one at the beginning and one at the end of the study. Figure 2 shows a map of the streets around the former premises of the Dutch National MS Foundation, Rotterdam, the Netherlands, on which the validation assessments took place, with an approximate path that was walked, reconstructed from one of the s2MWTs.

### Data Analysis

The distance walked during the s2MWT was calculated with a proprietary algorithm that applied a path reconstruction on the GPS data. This algorithm was tested on more than 10 different smartphones and optimized using various data sets held by the app manufacturer. Among the constraints applied on the reconstructed path is that it has to be possible to walk the path in 2 minutes.

In the data cleaning process, smartphone walking tests were removed when the data collected in a test were considered to be of low quality: (1) s2MWT duration <100 seconds; (2) s2MWT duration ≥140 seconds; (3) GPS data accuracy median ≥30 m; (4) GPS data accuracy SD ≥100 m; and (5) calculated distance walked <10 m.

For the test-retest analysis, we compared successive s2MWTs that were left after data cleaning and were less than 20 days apart.

### Statistical Analysis

Unless mentioned otherwise, the statistical analysis was performed using SciPy (version 1.2.1) in combination with Python (version 3.6.7). Shapiro-Wilk tests were used to check if variables were normally distributed. If they were not, visual inspection led to the removal of at most 2 outliers, after which a Shapiro-Wilk test confirmed a normal distribution. Therefore, the statistical tests mentioned could be applied. However, for the calculation of the Spearman rank correlations, we did not need to remove any outliers, as normality of the distributions is not a requirement for this calculation. *P* values <.05 were considered statistically significant for all statistical tests.

Two-sided *t* tests were conducted for the null hypothesis stating that the distance walked on the first s2MWT by persons with MS and distance walked by HC participants in both control groups have identical average (expected) values. A 2-sample Kolmogorov-Smirnov test was applied to the distributions of distance walked on the first s2MWT of the HC-matched group and HC-normative group to investigate if the 2 groups of HC participants had the same underlying distribution.

To investigate the concurrent validity of the s2MWT, we (1) conducted 2-sided *t* tests between the 2MWT distance and the s2MWT distance measured in the validation experiments; (2) calculated the Spearman rank correlation between the 2MWT distance and the s2MWT distance; (3) calculated the Spearman rank correlation between the EDSS score and the s2MWT distance; and (4) calculated the intraclass correlation coefficient (ICC) between the 2MWT distance and the s2MWT distance using a 2-way mixed effects model on absolute agreement for a single measurement: the ICC(A,1), following the nomenclature of McGraw et al [21]. The ICCs were calculated using the R package *irr* version 0.84.1 in combination with R version 3.5.1. ICC values <0.20 were considered *poor*, values between 0.20 and 0.39 were considered *fair*, values between 0.40 and 0.59 were considered *moderate*, values between 0.60 and 0.79 were considered *good*, and values >0.80 were considered *very good* [22,23]. In all 3 analyses of the concurrent validity, we corrected for the fact that there were (at most) 2 validation assessments per user by replacing the individual observations by the subject mean [24].

Test-retest reliability was determined by calculating the ICC(A,1) between measurements at different times. We used the same acceptance criteria for the test-retest reliability ICCs as for the concurrent validity ICCs. These ICC values were used in combination with the pooled SD of the test and retest to determine the SEM and the smallest detectable change (SDC), using the formulas [25]:



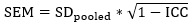





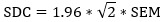



Internal consistency was evaluated and quantified using Cronbach α, in which α>.7 was defined to be acceptable [26,27]. The effect size, as measured by Cohen *d*, was determined to investigate the practice effect. Cohen *d* values <0.20 were considered small, values between 0.20 and 0.50 were considered medium, and values between 0.50 and 0.80 were considered large [28,29].

## Results

### Participant Demographics

The mean, median, and SD in the ages for the various groups are listed in Table 1, including the gender; number of participants in each education category (for the HC-normative group, no education information was collected); and the mean, median, and SD in the EDSS score (persons with MS). Even though having an EDSS score between 1.5 and 6.5 was one of the inclusion criteria, 1 person with MS with an EDSS score of 0 was enrolled in the study.

**Table 1:** Participant demographics.

Characteristics			Patients with relapsing-remitting multiple sclerosis (n=25)		HC^a^ matched (n=21)		HC normative (n=58)
**Age (years)**							
	Values, mean (SD)	40 (8)		37 (8)		34 (8)	
	Values, median	43		36		32	
**Gender**							
	Female, n	23		17		29	
	Male, n	2		4		29	
**Expanded Disability Status Scale**							
	Scores, mean (SD)	3.1 (1.4)		N/A^b^		N/A	
	Scores, median	3.0		N/A		N/A	
**Years since diagnosis**							
	Values, mean (SD)	6 (4.4)		N/A		N/A	
	Values, median	4		N/A		N/A	

^a^HC: healthy control.

^b^N/A: not applicable.

### Adherence and Number of Tests Done

In total, 104 study participants completed the assessments as scheduled, whereas 2 participants did not complete the study (1 person with MS and 1 HC participant in the HC-matched group). However, the validation assessments they did on the first day of the study could still be used to answer some of our research questions. The person with MS that dropped out of the study had an EDSS score of 3.5.

In total, 755 s2MWTs were done, which is 121 more than planned in the study protocol. This is because 91% (42/46) of the persons with MS and HC participants in the matched group continued to perform the tests after completing the 4 weeks of self-monitoring that was planned in the study protocol. This resulted in 72% (33/46) of these study participants completing at least 10 s2MWTs, the number that was planned in the study protocol, although mostly not with 3 days in between tests. The other 28% (13/46) of the persons with MS and HC participants in the matched group completed a total of 95 s2MWTs, which is, on average, more than 7 (SD 1.6) s2MWTs per person. In total, one could say that there was an s2MWT adherence rate of 92.4% (425/460) in these 2 groups. For the HCs in the normative group, who had a light study scheme with 3 s2MWTs in total, we only included participants who completed all 3 s2MWTs; therefore, we could not determine the adherence rate for this group.

One or more of the filtering criteria mentioned in the Data Analysis section was met in 23.3% (176/755) of all s2MWTs that were completed. The data loss rates were 23.6% (87/369) for the persons with MS, 26.4% (56/212) for the participants in the HC-matched group, and 19% (33/174) for participants in the HC-normative group. In total, 15.8% (119/755) of the assessments were filtered out because the duration of the s2MWT was <100 or >140 seconds, 7.2% (54/755) of the assessments were filtered out because the GPS accuracy was too low, and 0.4% (3/755) of the assessments were filtered out because the predicted distance walked was ≤10 m.

**Figure 3:**
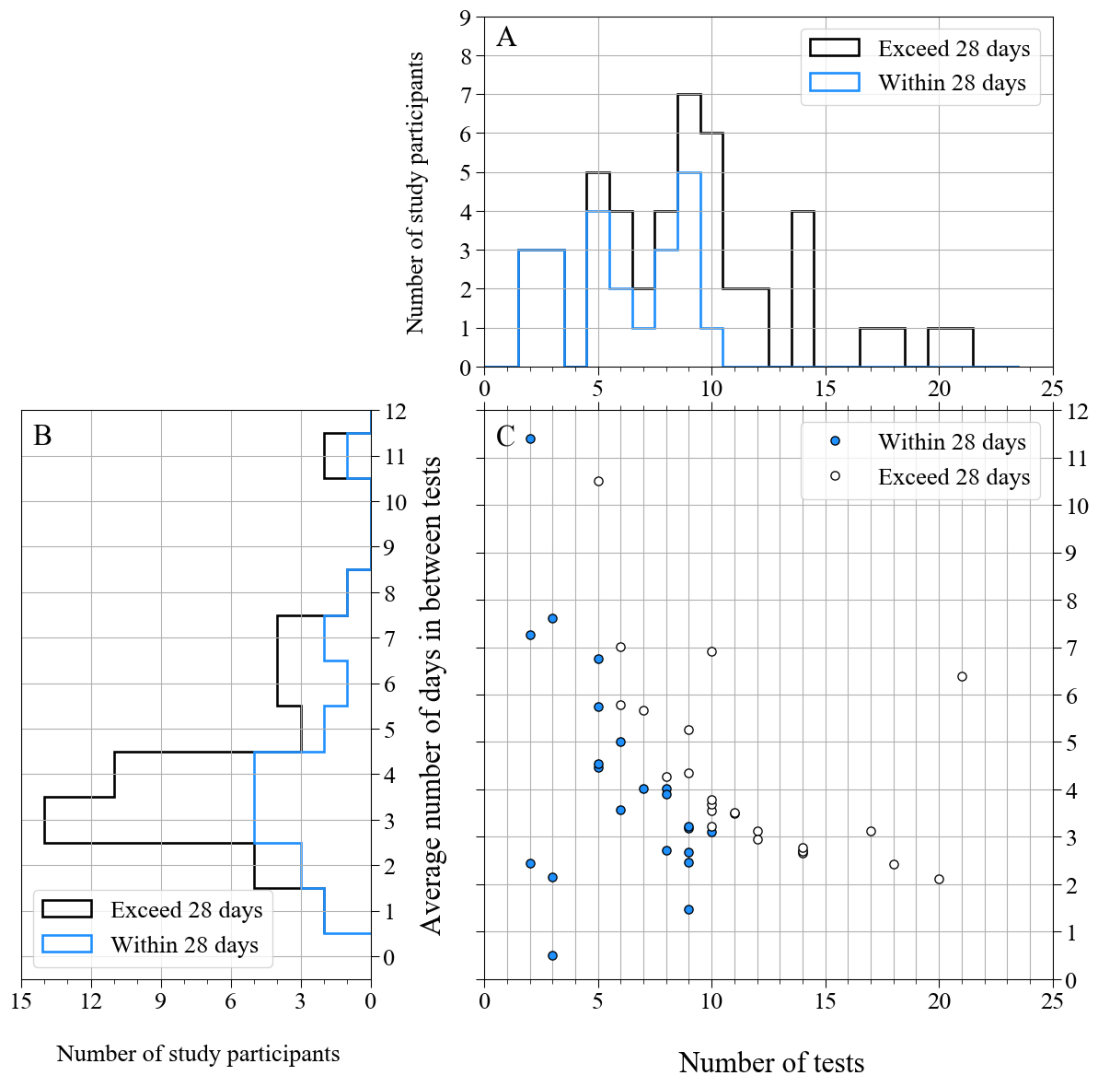
(A) Histogram showing the distribution of the number of smartphone 2-Minute Walking Tests per study participant that remained after data cleaning and was used to determine the test-retest reliability. (B) Histogram showing the distribution of the average number of days in between these tests per study participant. (C) Scatter plot that shows the relation between the number of tests (horizontal axis) and the average number of days in between these tests (vertical axis) for all study participants. Filled circles correspond to participants whose tests were done within 28 days, and open circles correspond to those whose tests were done in more than 28 days. The blue lines in panels A and B correspond to these filled circles, and the black lines in panels A and B correspond to the open circles.

Figure 3 shows the distribution of the number of s2MWTs performed by the persons with MS and HC participants that was left after data cleaning, the distribution of the average number of days between the tests per person with MS or HC participant in the matched group, and the relation between the number of tests and the average number of days in between tests in these groups.

Even though there were more than 10 s2MWTs left after data cleaning that could be used to determine the test-retest reliability for 26% (12/46) of the persons with MS and HC participants in the matched group, we included at most the first 10 assessments for the test-retest reliability calculations, as planned in the study protocol. After data cleaning, 43.8% (160/365) of the consecutive s2MWTs were between 2 and 4 days apart, and 60.5% (221/365) were between 1 and 5 days apart. For 52% (24/46) of the persons with MS and HC participants in the matched group, the timespan in which tests were completed exceeded the 4-week period that was planned in the study protocol.

There were missing smartphone data for 2 validation assessments on the first day of the study because of technical issues with the app, and 2 other validation assessments had missing smartphone data because of issues with the smartphone. Moreover, 1 study participant was not able to perform the validation assessment on the last day of the study because of the severity of MS; she had an EDSS score of 6.5 and walked <25 m on the first validation assessment.

In the cleaning process, 35% (31/89) of the validation assessments were removed, including 1 of the 2 validation assessments of the dropouts because at least one of the filtering criteria mentioned in the Data Analysis section was met. We averaged the distance walked in the 2 validation assessments of a study participant when both validation assessments of this study participant remained in our sample after data cleaning, as mentioned in the Statistical Analysis section. This resulted in 43 pairs of distance determinations (2MWT-s2MWT).

### Distinction Between Persons With MS and HCs

**Figure 4:**
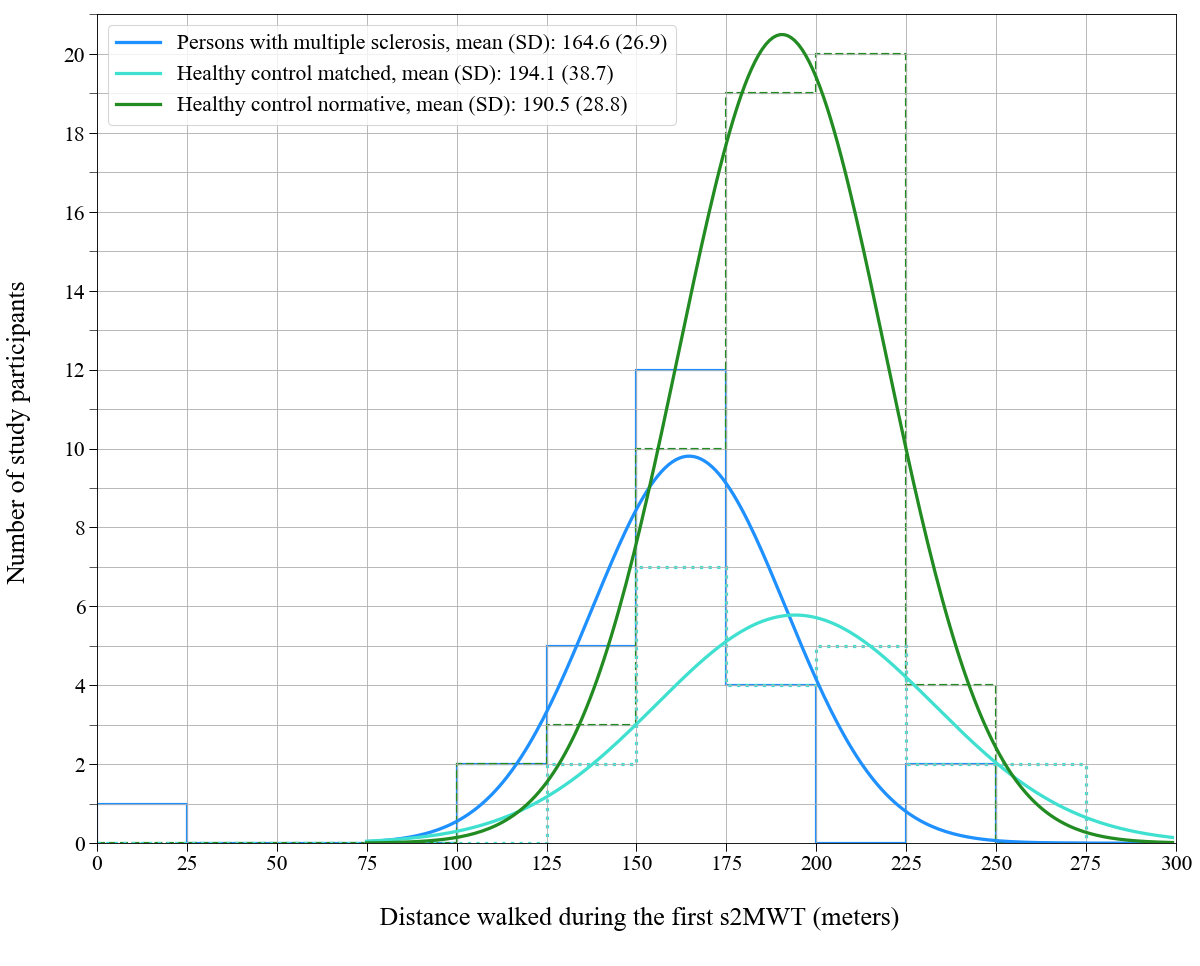
Distributions of the distance walked on the first smartphone-based 2-Minute Walking Test (s2MWT) for the 3 groups in this study. The thin solid line shows the distribution for persons with multiple sclerosis, the dotted line shows the distribution for the healthy control–matched group, and the dashed line shows the distribution for the healthy control–normative group. The thick solid lines represent Gaussian fits to the distributions, of which the means (SDs) are shown in the legend.

Figure 4 shows the distance walked during the first valid s2MWT in the persons with MS and in the 2 control groups. Because one person with MS (with an EDSS score of 6.5) walked <25 m, the distribution of distances walked by the persons with MS was only normally distributed after removal of this data point (*P*=.06 on a Shapiro-Wilk test). The estimated distance walked was normally distributed for the HC-matched group and the HC-normative group because the Shapiro-Wilk tests on these distributions yielded *P*=.48 and *P*=.24, respectively.

The 2 groups of HC participants had the same underlying distribution as confirmed using a 2-sample Kolmogorov-Smirnov test (Kolmogorov-Smirnov statistic=0.21; *P*=.44). Independent 2-sample *t* tests between persons with MS versus the participants in the HC-matched group and persons with MS versus the participants in the HC-normative group confirmed that the s2MWT can distinguish between persons with MS and HC participants at the group level (*P*=.004 and *P*<.001, respectively).

### Concurrent Validity

**Figure 5:**
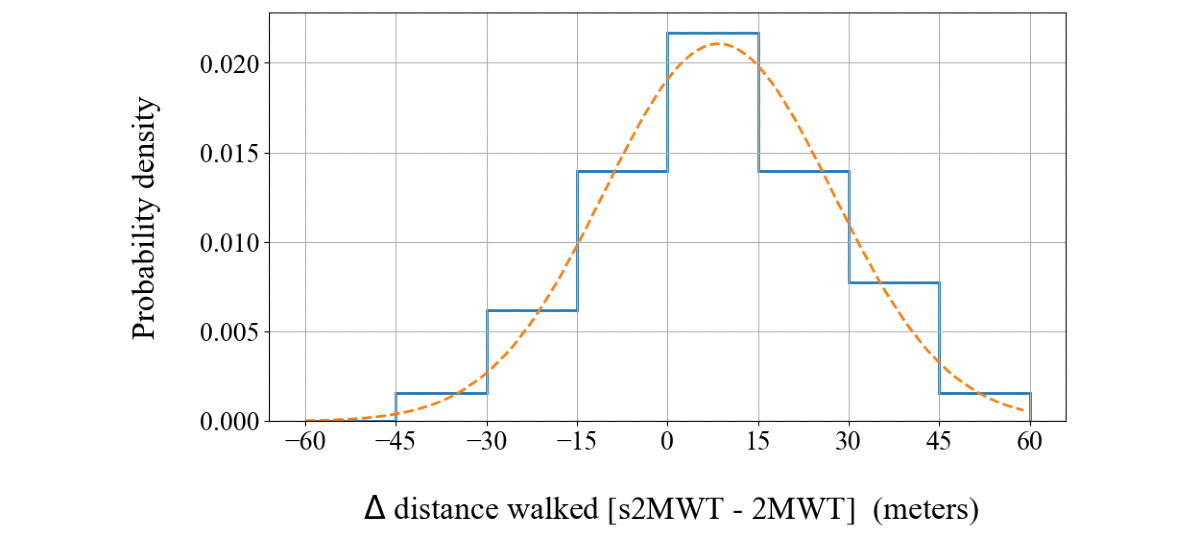
Distribution of differences between the measured distance walked by the app (smartphone-based 2-Minute Walking Test [s2MWT]) and the distance markers (2-Minute Walking Test [2MWT]). The dashed line represents a normal distribution.

A Shapiro-Wilk test on the distribution of the differences between the distance determined with the s2MWT and the distance measured using distance markers on the pavement accepted normality (*P=*.97). This distribution is shown in Figure 5. The distance determined with the s2MWT was on average 8.43 m or 5% (SD 18.9 m or 11%) higher than the distance on the 2MWT (n=43). Here, the percentage is the difference divided by the 2MWT distance, times 100.

A dependent 2-sided *t* test between the 2MWT distance and the s2MWT distance measured in these validation assessments yielded a test statistic of −2.9, with *P*=.006. Because this is below the significance level of 0.05, we must reject the null hypothesis of equal averages.

**Figure 6:**
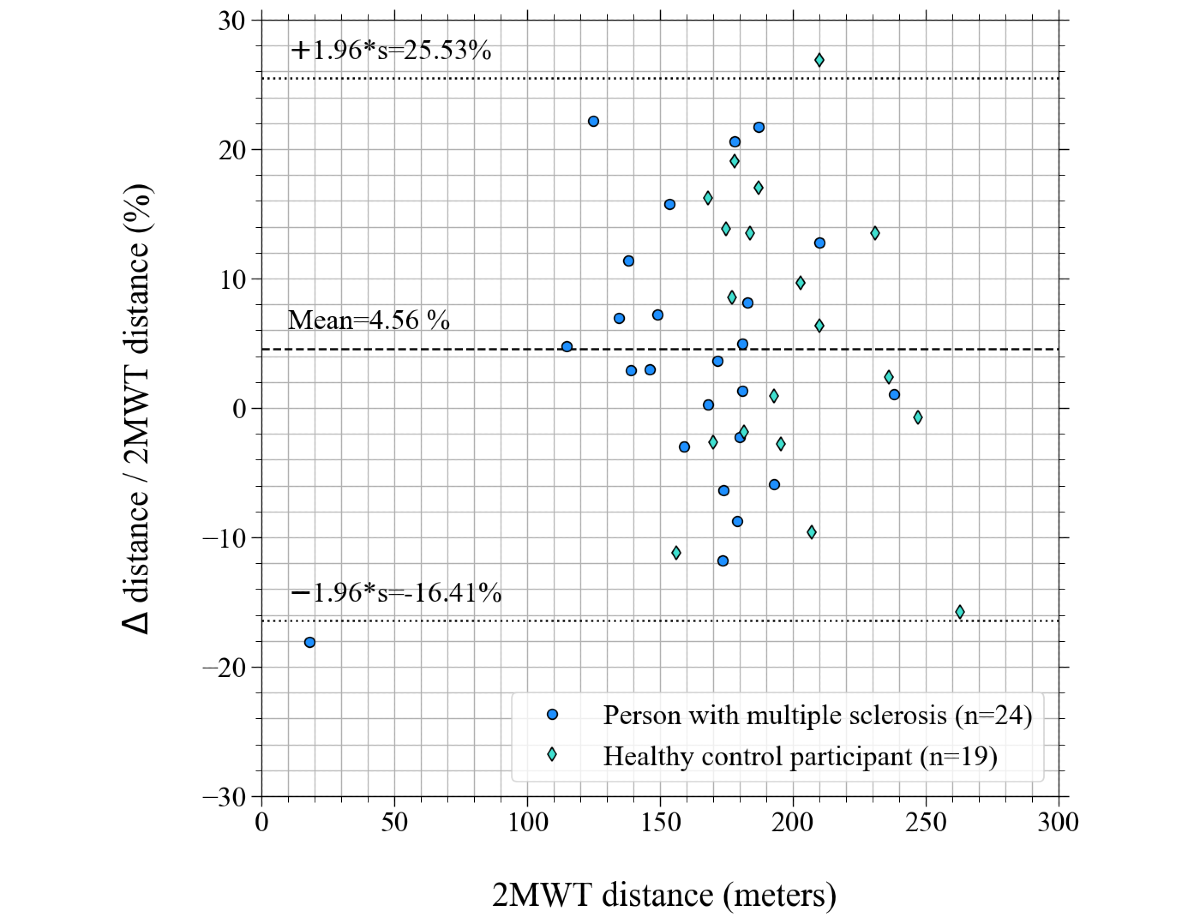
Bland Altman plot of differences between the measured "distance walked" by the app (smartphone-based 2-Minute Walking Test distance) and the distance markers (2-Minute Walking Test distance) expressed as percentages of the 2-Minute Walking Test distance (∆ distance / 2-Minute Walking Test distance) versus the 2-Minute Walking Test distance. The dashed line shows the mean percentage difference, the dotted lines show the 95% CI.

Figure 6 is a Bland-Altman plot of the 43 pairs of distance determinations derived from the validation assessments. The 2MWT distance is shown on the horizontal axis. On the vertical axis of this plot, the percentage difference between the s2MWT and the 2MWT is shown, which is calculated as explained above. The mean percentage difference is shown as a horizontal dashed line, the 95% CI, at 1.96 × SD around the mean difference is presented with 2 horizontal dotted lines. Distance determinations of persons with MS are presented with blue circles and those of HCs in the matched group, with green diamonds.

**Figure 7:**
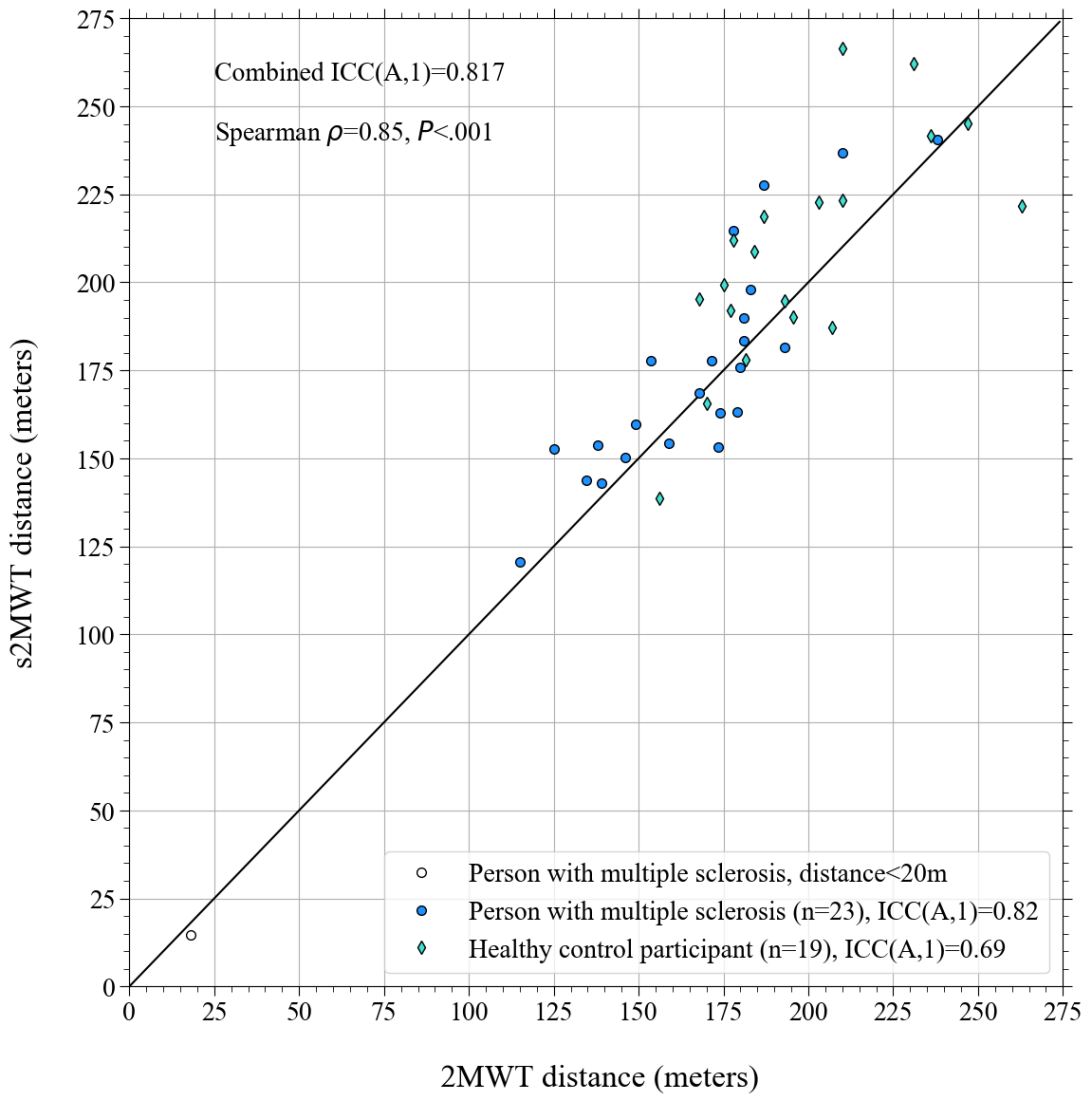
Scatter plot to show the ICC(A,1) values and the correlation (Spearman rho, upper left corner) between the measured ‘distance walked’ using distance markers (2-Minute Walking Test, horizontal axis) and the distance measured by the app (smartphone-based 2-Minute Walking Test, vertical axis). A 45 degree black solid line shows a 1:1 correlation.

In Figure 7, the distance measured on the s2MWT is plotted against the distance measured on the 2MWT. ICC(A,1) values are shown for persons with MS and HCs separately (see legend in Figure 7) and for the combined data set, which is shown in the top-left corner of Figure 7. The Spearman rank correlation and corresponding *P* value for the combined data set are also shown in the top-left corner of Figure 7.

**Figure 8:**
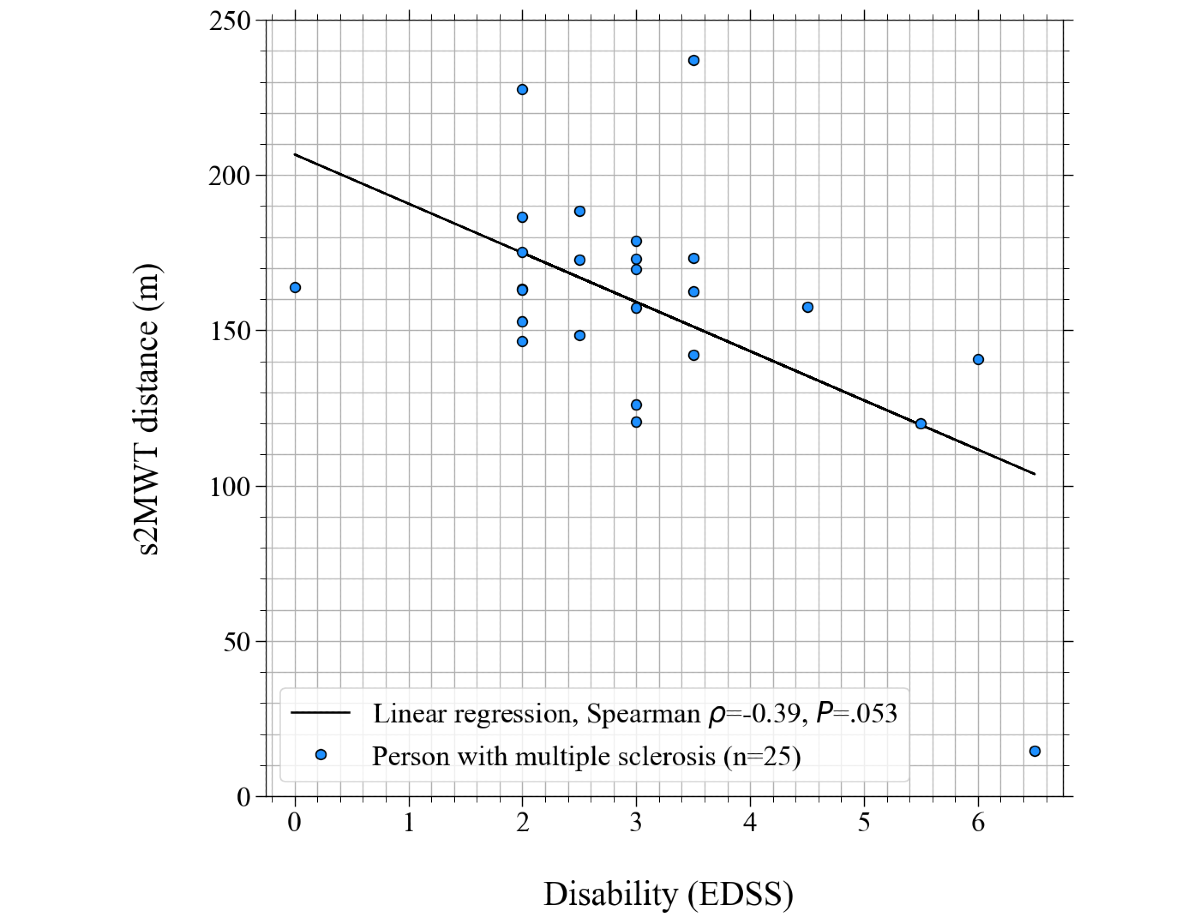
Relation between the distance walked on the first test done with the smartphone-based 2-Minute Walking Test and the Expanded Disability Status Scale score, for the 25 persons with multiple sclerosis that participated in this study.

The relation between the disability as measured by the EDSS and the s2MWT scores is presented in Figure 8. Distance determinations of persons with MS are presented with blue circles. A black solid linear regression line is overplotted. The Spearman rank correlation and corresponding *P* values between the two variables are also shown in the bottom-left corner of Figure 8. We found a fair correlation that however failed to be statistically significant.

### Test-Retest Reliability

The ICCs(A,1) (with their 95% CI), Cronbach α, Cohen *d*, SEM, and SDC values that were derived from 9 test-retests of the s2MWT for the persons with MS and the participants in the HC-matched group are listed in Table 2, and the values of those derived from the 2 test-retests performed by the HC-normative group are shown in Table 3. The mean values of the ICC(A,1) for persons with MS, participants in the HC-matched group, and participants in the HC-normative group were 0.649 (SD 0.150), 0.600 (SD 0.090), and 0.700 (SD 0.029), respectively. These indicate a good test-retest reliability. The corresponding average SDC values were 58.1 (SD 13.7) m, 61.8 (SD 15.0) m, and 51.1 (SD 0.1) m, which is 35% (58.1/165.5), 32% (61.8/193.5), and 27% (51.1/188.7) of the group’s mean distance walked during test and retest, respectively.

**Table 2:** Test-retest reliability scores of the smartphone-based 2-Minute Walking Test for persons with multiple sclerosis and participants in the healthy control–matched group.

Test-retest	ICC^a^(A,1; 95% CI) of persons with MS^b^	ICC(A,1; 95% CI) of HC^c^ participants	Cronbach α of persons with MS	Cronbach α of HC participants	Cohen *d* of persons with MS	Cohen *d* of HC participants	SEM of persons with MS (m)	SEM of HC participants (m)	SDC^d^ of persons with MS (m)	SDC of HC participants (m)
1	0.462 (0.086 to 0.727)	0.554 (0.171 to 0.791)	.640	.708	0.301	0.150	24.0	30.6	66.4	84.7
2	0.509 (0.142 to 0.759)	0.589 (0.206 to 0.818)	.694	.742	0.369	0.219	24.0	28.6	66.6	79.3
3	0.624 (0.280 to 0.827)	0.723 (0.391 to 0.890)	.768	.863	0.185	0.145	23.2	17.6	64.2	48.8
4	0.657 (0.322 to 0.845)	0.439 (−0.057 to 0.756)	.787	.596	0.098	0.046	23.3	24.0	64.6	66.5
5	0.793 (0.547 to 0.913)	0.545 (−0.008 to 0.837)	.880	.690	0.035	0.066	15.9	23.0	44.2	63.7
6	0.873 (0.698 to 0.950)	0.659 (0.141 to 0.895)	.932	.788	0.114	0.198	12.3	23.2	34.1	64.4
7	0.823 (0.584 to 0.930)	0.633 (−0.031 to 0.905)	.912	.757	0.242	0.120	15.5	21.3	42.9	59.1
8	0.504 (0.059 to 0.781)	0.707 (−0.447 to 0.966)	.663	.794	0.154	0.010	25.5	12.8	70.6	35.4
9	0.595 (0.148 to 0.846)	0.553 (−0.773 to 0.986)	.761	.699	0.360	0.615	25.1	19.6	69.9	54.4
Mean	0.649 (0.318 to 0.842)	0.600 (−0.045 to 0.871)	.782	.734	0.206	0.174	21.0	22.3	58.1	61.8

^a^ICC: intraclass correlation coefficient.

^b^MS: multiple sclerosis.

^c^HC: healthy control.

^d^SDC: smallest detectable change.

**Table 3:** Test-retest reliability of the smartphone-based 2-Minute Walking Test for the healthy control–normative group.

Test-retest	ICC^a^ (A,1; 95% CI)	Cronbach α	Cohen *d*	SEM (m)	Smallest detectable change (m)
s2MWT test-retest 1	0.721 (0.548-0.835)	.835	0.031	18.4	51.0
s2MWT test-retest 2	0.680 (0.421-0.840)	.807	0.119	18.4	51.1
Mean	0.700 (0.480-0.837)	.821	0.075	18.4	51.1

^a^ICC: intraclass correlation coefficient.

We also calculated the ICC(A,1), Cronbach α, Cohen *d*, SEM, and SDC corresponding to the comparison of the average of the first 5 s2MWTs with the second 5 s2MWTs for persons with MS and the matched HCs. The results are shown in Table 4. When averaged over 5 tests, the SDC values for persons with MS and the matched HCs were reduced to 16% (25.1/156.5) and 21% (40.7/193.5) of the group’s mean distance walked, respectively.

**Table 4:** Test-retest reliability scores of the smartphone-based 2-Minute Walking Test based on the comparison of the mean value of the first 5 test scores with the mean value of the second 5 test scores for persons with multiple sclerosis and the healthy control participants in the matched group.

Group	ICC^a^ (A,1; 95% CI)	Cronbach α		Cohen *d*	SEM (m)	Smallest detectable change (m)
Persons with multiple sclerosis	0.956 (0.898-0.981)	.977	0.028		9.0	25.1
Healthy control–matched	0.750 (0.381-0.915)	.859	0.220		14.7	40.7

^a^ICC: intraclass correlation coefficient.

The Cohen *d* values in Tables 2, 3, and 4 show that the practice effect is medium for persons with MS and small for HCs in the matched group when averaged over all test-retests. However, when comparing the average scores of the first 5 tests with those of the second 5 tests, the practice effect is small for persons with MS and medium for HCs in the matched group. For the HCs in the normative group, practice effects were small. When investigating the practice effect in the validation assessments, we found, on average, an increase of 10.6% (SD 12.5%) and 9.7% (SD 7.8%) in 2MWT distance walked for persons with MS and distance walked by HC participants, respectively. With corresponding Cohen *d* values of 0.66 (persons with MS) and 0.59 (HC-matched participants), this is considered a large practice effect.

### Interview Results

Out of 7 participants with MS, 5 participants with MS who were interviewed about their experiences with the smartphone app and Fitbit activity tracker in general experienced some technical difficulties with the test, such as issues with the GPS signal or that the test suddenly stopped. Respondents were frustrated about these issues. Furthermore, 3 respondents mentioned that they had more or less stable s2MWT results during the study, whereas for 4 respondents, the results showed more fluctuation. One respondent explicitly stated that being faced with these fluctuations was emotionally confronting to her. Moreover, 3 respondents experienced a competitive element in the s2MWT—feeling the pressure to reach the same distance every time. Of these, 1 respondent wanted to *improve* her score by walking a little faster. Difficulties in making the s2MWT a routine element of their daily life were reported by 3 respondents. For instance, 1 respondent expressed annoyance about having to perform the test at specific moments of the week, whereas another respondent sometimes forgot to do the test, which made her feel guilty. However, in general the interview respondents expressed that the instructions for the s2MWT were clear and that the test was easy to perform.

## Discussion

### Principal Findings

We found that the s2MWT can distinguish between persons with MS and HC participants at the group level. The estimated distance walked on the s2MWT is, on average, 5% (SD 11%) higher than the distance measured using distance markers. Therefore, we cannot conclude that the average s2MWT distance is equal to the average 2MWT distance, which is also reflected in the outcome of a dependent 2-sided *t* test between the 2 distributions (*P*<.05). The duration of the s2MWTs of the persons with MS and matched HCs was, on average, 2.86 (SD 5.74) seconds too long, which could explain why the s2MWT overestimates the distance walked. However, the distances measured with the s2MWT highly correlated with the 2MWT distances; we found a Spearman rank correlation of 0.85. Furthermore, a very good concurrent validity for persons with MS and a good concurrent validity for HC participants was established, given the ICC(A,1) values of 0.82 and 0.69 between the s2MWT distance and the 2MWT distance, respectively.

The correlation between the s2MWT score and disability as measured by the EDSS was fair but not significant. However, per the definition of the EDSS, persons with MS having EDSS scores of 4 or lower have full ambulation (including the ability to walk without aid or rest for some 500 m) [3]. As there were only 4 study participants with EDSS scores >4, it is not strange that the correlation that we found was not significant. Furthermore, if we had not included the person with the EDSS score of 0, we would have found a significant (*P*=.049) Spearmen rank correlation of −0.41 between EDSS and the distance walked on the first s2MWT.

The 9 test-retest analyses showed that the test-retest reliability was good for the persons with MS (average ICC[A,1] 0.649, SD 0.150) and matched HCs (average ICC[A,1] 0.600, SD 0.090). In addition, the test-retest reliability we found in the HC-normative group was good, with an average ICC(A,1) value of 2 test-retest analyses of 0.700 (SD 0.029). Practice effects between consecutive tests were, on average, medium for persons with MS and small for HCs in the matched group. However, the practice effect between the average score of the first 5 s2MWTs compared with the average score of the second 5 s2MWTs was small for persons with MS and medium for HCs in the matched group. The practice effect between the 2 validation assessments was large. The study participants may have felt pressure to reach the same distance as during the first validation assessment, which could have resulted in faster walking speeds during the second validation assessment. On average, there were 65 (SD 44) days in between the 2 validation assessments.

We derived an SDC of 58.1 m and 61.8 m for persons with MS and matched HCs, respectively, from the individual s2MWT test-retest assessments. These values seem rather large given the respective typical walked distances of 165 m and 194 m of these groups. Large day-to-day variations in the distance walked during the s2MWT and the s2MWT measurement error contributed to the large SDC. However, a smaller SDC can be obtained by averaging over multiple measurements. Indeed, from comparing the average distance walked in the first 5 s2MWTs with the average distance walked in the second 5 s2MWTs, we derived SDC values of 25.1 m and 40.7 m for persons with MS and matched HCs, respectively. This finding implies that changes in walking speed that result in a more than 25.1 m change on the s2MWT score, when averaged over 5 measurements, are above day-to-day variations and measurement noise.

It is important to distinguish the SDC from the minimally important clinical difference (MICD). The MICD is the smallest change in test score that is perceived as important by patients and clinicians, whereas the SDC is the smallest change that can be detected beyond measurement error. Preferably, a measurement instrument has an SDC that is smaller than the MICD, so that all clinically relevant changes can be distinguished from measurement error. For the 2MWT, a MICD of <10 m was found after MS rehabilitation [30], but a change of 20% is generally considered an MICD for the T25FW [31]—another walking test that is often used in MS—that highly correlates with the 2MWT [6]. Although the SDC that we find is larger than 10 m, the 25.1 m that we found after averaging 5 measurements correspond to 16% of the group’s mean distance walked, which is less than 20%.

The interview respondents shared some important considerations. Aside from technical issues that need to be solved, performing the test can evoke emotional responses. For instance, patients can feel confronted by the test results, experience pressure to reach a certain distance, or feel annoyed by having to perform the test regularly. However, the interview respondents also expressed that the instructions for the s2MWT were clear and that the test was easy to perform. Furthermore, in clinical practice, the s2MWT is expected to be scheduled less often than every 3 days, which was the test frequency applied in this study. If the s2MWTs are scheduled, for example, weekly, always at the same day of the week and at the same time of the day chosen by the person with MS, it should be easier to make self-monitoring walking speed a routine element of daily life.

### Limitations and Future Work

The 2MWT is a relevant outcome measure not only for persons with MS but in a variety of health conditions such as chronic obstructive pulmonary disease [32], lower limb amputation [33], cardiovascular disease [34], osteoarthritis [35], Parkinson disease [36], and Alzheimer disease [37]. This study limits itself to persons with MS, and the validity of the s2MWT for applications outside MS will still have to be demonstrated. Furthermore, 84% (21/25) of the persons with MS participating in this study had an EDSS score below 4. As can be seen in Figures 5 and 6, all persons with MS walked ≥100 m in the validation assessments, apart from 1 study participant (who had an EDSS score of 6.5; Figure 8). Therefore, this validation of the s2MWT limits itself to persons with MS who are able to walk ≥100 m in 2 minutes. The validity of the s2MWT for slow walkers, that is, those who walk ≤100 m in 2 minutes, has to be demonstrated in a different validation study.

For this analysis, we had to limit ourselves to 76.7% (579/755) of the s2MWT assessments done during MS Self. Most assessments that were filtered out did not pass the acceptance criteria that the s2MWT duration should be 120 seconds within a 20-second margin. The assessment duration is derived from the time difference between the first and the last GPS data point. The GPS data collection frequency is typically 1 data point per second or even per 2 seconds; therefore, the s2MWT assessment duration is often 1 or sometimes even 2 seconds ≤2 minutes. s2MWT durations that have much larger deviations from 120 seconds than 2 seconds must have been the result of data collection deficiency. This data collection deficiency may have arisen from memory loss or a clock synchronization error when the app was unintentionally running in the background. The 20-second margin that we considered acceptable here is far from ideal. Assessments that are on the border of being acceptable (eg, an assessment with a duration of 105 seconds or an assessment with a duration of 139 seconds) are expected to result in a poor estimation of the distance walked in 2 minutes. The data collection during s2MWT assessments was improved after this study, and the improved s2MWT is currently implemented in the MS sherpa app. It is expected that this new s2MWT has a higher concurrent validity and test-retest reliability than the s2MWT that was used in this study. Preliminary results from the APPS MS (Assessing fatigue, disease activity and Progression through smartPhone Surveillance in Multiple Sclerosis) study confirm that the test-retest reliability has indeed improved [18].

One should keep in mind that the s2MWT was performed on the study participant’s phone, and technical specifications, in particular GPS accuracy, may not be equivalent for all devices. Although this study was not set up to compare s2MWT performances of different phone types, we have not found large differences between phone types in preclinical studies. Here, we have partially accounted for the variability in phone types in the data cleaning process, where we filtered out assessments with low GPS accuracy.

Another limitation of this study and an opportunity for future improvements is the algorithm that was used to calculate the distance walked. The version used for this paper makes use of the GPS data alone, while accelerometer data was also collected during the s2MWTs in this study. GPS gives information about where a person has walked, while the accelerometer provides information about the forces that are exerted on the mobile phone during the 2MWT, including gravity. The accelerometer comes from an inertial measurement unit that is embedded in the phone. Future work can improve the algorithm by making use of the accelerometer data and data from various other sensors of the inertial measurement unit, such as angular velocity from a gyroscope. These additional sources of data allow for various other kinds of information to be extracted, such as balance and gait characteristics [8-13]. Furthermore, inertial measurement unit data have been shown to be suitable for walking distance estimation, for example, using machine learning or dead reckoning [38,39]. The reason to prefer an alternative to a purely GPS-based solution is that GPS has inherent inaccuracies because of the dependency of satellites in orbit. Anything such as large buildings or overpasses can significantly reduce the accuracy of the GPS, resulting in inaccurate distance estimations. The strengths of both data sources combined can positively influence the reliability of the measurements.

In the s2MWT instructions during MS Self, it was not requested to walk the same path every time the test is done. If the test and retest were not done on the same location, the test result was likely to be affected because of environmental effects, for example, in an uphill walk, the user was expected to reach a shorter distance than in a flat walk. In additional analyses, we observed that walking a straight line was also preferable to reach more accurate distance calculations. Therefore, we improved the instructions for the users in the s2MWT that is currently implemented in the MS sherpa app—they should walk in a straight line as much as possible and try to walk the same route every time they do the test.

In an MS clinic, a 2MWT assessment outcome is typically established after averaging the distances walked in two or three 2MWTs. In this study, we scheduled one s2MWT at a time for the convenience of the persons with MS performing the assessments. Averaging over repeated smartphone assessments may also improve the accuracy of the test. This should also be kept in mind when comparing the test-retest reliability of various walking tests in the literature.

The 2MWT distance estimations in the validation assessments of MS Self may be up to a few meters off because of small deviations in the walked paths with respect to the path set out by the distance markers. In future studies that include validation experiments such as those in MS Self, we propose to use something akin to a measuring wheel, also known as a surveyor’s wheel, to determine the actual distance walked during a 2MWT by walking alongside the person being tested. We expect that the distance walked in real life settings can be determined very accurately with a measuring wheel. Furthermore, it would be easy to choose a different path for various validation assessments instead of following the same distance markers as closely as possible each time.

As mentioned in the Principal Findings section, there were, on average, 65 (SD 44) days in between the 2 validation assessments, and as shown in Figure 3, some participants had more than 130 days between the 2 study visits. Although this does not affect the smartphone test-retest reliability, as we only compared successive s2MWTs that were less than 20 days apart for each user, it should be kept in mind when interpreting the practice effect in the validation assessments. Furthermore, we did not investigate whether the persons with MS experienced any relapse during the follow-up nor did we investigate whether they took any new medication that could have influenced their walking performances, which would affect the test-retest reliability. However, we did register that the occurrence of a relapse was the reason to drop out of the study for one of the included persons with MS.

In a future study, we will investigate how frequently the s2MWT should be performed to obtain a clear picture of the walking speed of a person with MS over time. Furthermore, we will investigate how monitoring walking speed in a patients’ home environment could help in making clinical decisions, for example, in the evaluation of the effectiveness of a medicine used to improve walking ability in persons with MS, such as fampridine, or for measuring disease activity or disease progression in MS. One can imagine that this digital biomarker could help to early predict a transition to the secondary progressive phase or to detect suboptimal treatment response. Furthermore, this digital biomarker could potentially be used for evaluating of the effect of MS rehabilitation.

### Comparison With Previous Work

Although much of the literature is available on the 2MWT as a relevant tool to measure walking speed in persons with MS [6,40-44], little has been published about 2MWTs that can be self-administered on a smartphone. The Floodlight smartphone monitoring app for persons with MS, which was developed by Roche, contains an s2MWT, that was shown to moderately correlate with T25FW time [45]. This has recently also been shown for the s2MWT that is implemented in MS sherpa [18]. The MSCopilot smartphone monitoring app for persons with MS contains another alternative to self-assessed walking speed. However, results on the performance of this smartphone walking test have not been individually reported but only for the combined digital Multiple Sclerosis Functional Composite assessment scores [15].

It is already known that self-monitoring can evoke strong emotions and sentiments [46]. It is crucial to be aware of the potential burden of self-monitoring to patients, as a significant user burden leads to unwillingness to use these technologies [47]. Therefore, it is important to explore how digital self-monitoring tools could be developed in such a way that the burden is reduced [46,47].

### Conclusions

This study shows a good concurrent validity of the s2MWT because the ICCs(A,1) between the 2MWT and the s2MWT for persons with MS and HC participants were 0.82 and 0.69, respectively. The distance determined with the s2MWT is, on average, 4.56% (SD 10.7%) larger than the distance measured using distance markers on the pavement. It is expected that this can largely be attributed to the s2MWT assessment durations that were, on average, 2.86 (SD 5.74) seconds too long—an artifact of the s2MWT that was used in this study that is no longer present in the s2MWT implemented in MS sherpa. The s2MWT has a good test-retest reliability because the ICC(A,1) values averaged over all test-retests performed by the persons with MS and both groups of HCs were in the range 0.6-0.7. We conclude that the s2MWT can be used to measure the distance that persons with MS walk in 2 minutes in the outdoors near their home. Clinical studies and clinical practice can benefit from this, as it allows the collection of real-world evidence for interventions aimed to improve the walking speed of a person with MS. Furthermore, a frequent assessment of walking speed in the home environment of a person with MS may improve clinical decision-making because it provides health care providers with quantitative information about changes in their patients’ walking speed.

## Abbreviations

2MWT: 2-Minute Walking Test
6MWT: 6-Minute Walking Test
EDSS: Expanded Disability Status Scale
HC: healthy control
ICC: intraclass correlation coefficient
MICD: minimally important clinical difference
MS: multiple sclerosis
SDC: smallest detectable change
s2MWT: smartphone-based 2-Minute Walking Test
T25FW: Timed 25-Foot Walk
